# Augmented Reality Navigation in Craniomaxillofacial/Head and Neck Surgery

**DOI:** 10.1002/oto2.70108

**Published:** 2025-04-11

**Authors:** E. Brandon Strong, Anuj Patel, Alexander P. Marston, Cameron Sadegh, Jeffrey Potts, Darin Johnston, David Ahn, Shae Bryant, Michael Li, Osama Raslan, Steven A. Lucero, Marc J. Fischer, Marike Zwienenberg, Neha Sharma, Florian Thieringer, Christian El Amm, Kiarash Shahlaie, Marc Metzger, E. Bradley Strong

**Affiliations:** ^1^ Department of Otolaryngology–Head and Neck Surgery University of California, Davis Davis California USA; ^2^ Department of Neurological Surgery University of California, Davis Davis California USA; ^3^ Department of Plastic and Reconstructive Surgery University of Oklahoma Oklahoma City Oklahoma USA; ^4^ Department of Oral and Maxillofacial Surgery David Grant Medical Center Fairfield California USA; ^5^ Department of Radiology University of California, Davis Davis California USA; ^6^ Department of Biomedical Engineering University of California, Davis Davis California USA; ^7^ Department of Computer Science Technical University of Munich Munich Germany; ^8^ Clinic of Oral and Craniomaxillofacial Surgery University Hospital Basel Basel Switzerland; ^9^ Medical Additive Manufacturing (Swiss MAM) Research Group, Department of Biomedical Engineering University of Basel Basel Switzerland; ^10^ Department of Oral and Maxillofacial Surgery University Hospital Freiburg Freiburg Germany

**Keywords:** augmented reality, augmented reality navigation, bilateral sagittal split operation, bimaxillary advancement, computer‐aided surgery, cranial vault remodeling, craniofacial, craniomaxillofacial surgery, extended reality, facial plastic reconstructive, fibula free flap, frontal sinus, fronto‐orbital advancement, hamartoma, head and neck cancer, Le Fort, mandible, mandibular distraction, mandibular reconstruction, meningioma, midface, mixed reality, naso‐orbito‐ethmoid, navigation, orbit, orthognathic, panfacial, spheno‐orbital, surgically assisted rapid palatal expansion (SARPE), trauma, tumors, virtual reality, virtual surgical planning, zygoma, zygomaticomaxillary

## Abstract

**Objective:**

This study aims to (1) develop an augmented reality (AR) navigation platform for craniomaxillofacial (CMF) and head and neck surgery; (2) apply it to a range of surgical cases; and (3) evaluate the advantages, disadvantages, and clinical opportunities for AR navigation.

**Study Design:**

A multi‐center retrospective case series.

**Setting:**

Four tertiary care academic centers.

**Methods:**

A novel AR navigation platform was collaboratively developed with Xironetic and deployed intraoperatively using only a head‐mounted display (Microsoft HoloLens 2). Virtual surgical plans were generated from computed tomography/magnetic resonance imaging data and uploaded onto the AR platform. A reference array was mounted to the patient, and the virtual plan was registered to the patient intraoperatively. A retrospective review of all AR‐navigated CMF cases since September 2023 was performed.

**Results:**

Thirty‐three cases were reviewed and classified as either trauma, orthognathic, tumor, or craniofacial. The AR platform had several advantages over traditional navigation including real‐time 3D visualization of the surgical plan, identification of critical structures, and real‐time tracking. Furthermore, this case series presents the first‐known examples of (1) AR instrument tracking for midface osteotomies, (2) AR tracking of the zygomaticomaxillary complex during fracture reduction, (3) mandibular tracking in orthognathic surgery, (4) AR fibula cutting guides for mandibular reconstruction, and (5) integration of real‐time infrared visualization in an AR headset for vasculature identification.

**Conclusion:**

While still a developing technology, AR navigation provides several advantages over traditional navigation for CMF and head and neck surgery, including heads up, interactive 3D visualization of the surgical plan, identification of critical anatomy, and real‐time tracking.

Craniomaxillofacial (CMF) surgery is challenging due to the complex 3‐dimensional (3D) anatomy and the limited exposure provided by minimally invasive incisions. Historically, presurgical planning has utilized flat panel, 2‐dimensional (2D) computed tomography (CT) imaging. For this planning, a surgeon would need to create a 3D mental model of the pathology and surgical plan, with the frequent need for improvisation in the operating room. Recent advances in presurgical planning software and custom implant design have allowed more planning to be done with computational models. However, the majority of intraoperative decision‐making still relies heavily on the surgeon's 3D mental modeling and spatial cognition.

In the early 2000s, the concept of computer‐aided surgery (CAS) gained significant traction.^1^ Many new tools were developed, including 3D visualization software,^2^ 3D‐printed models/guides/implants,^3^ intraoperative CT,^4^ and intraoperative navigation.^5^ The authors have previously described a CAS workflow, which divides CMF reconstruction into 3 phases: *virtual planning*, *surgical execution*, and *anatomic verification*.^1^ The most recent addition to this workflow is *extended reality* (XR) (Figure 1). XR is an umbrella term that includes both *virtual reality* (VR) and *augmented reality* (AR). VR utilizes a head‐mounted display (HMD) that covers the user's eyes, blocking the visualization of the physical environment and immersing the user in a computer‐generated virtual environment. AR uses a see‐through display, allowing users to interact with each other, virtual objects, and the environment.^6^ VR has been used extensively for virtual surgical planning,^7^, ^8^, ^9^, ^10^ while AR has been the primary choice for surgical execution and anatomic verification due to increased visibility of the surgical field.^11^, ^12^

**Figure 1:**
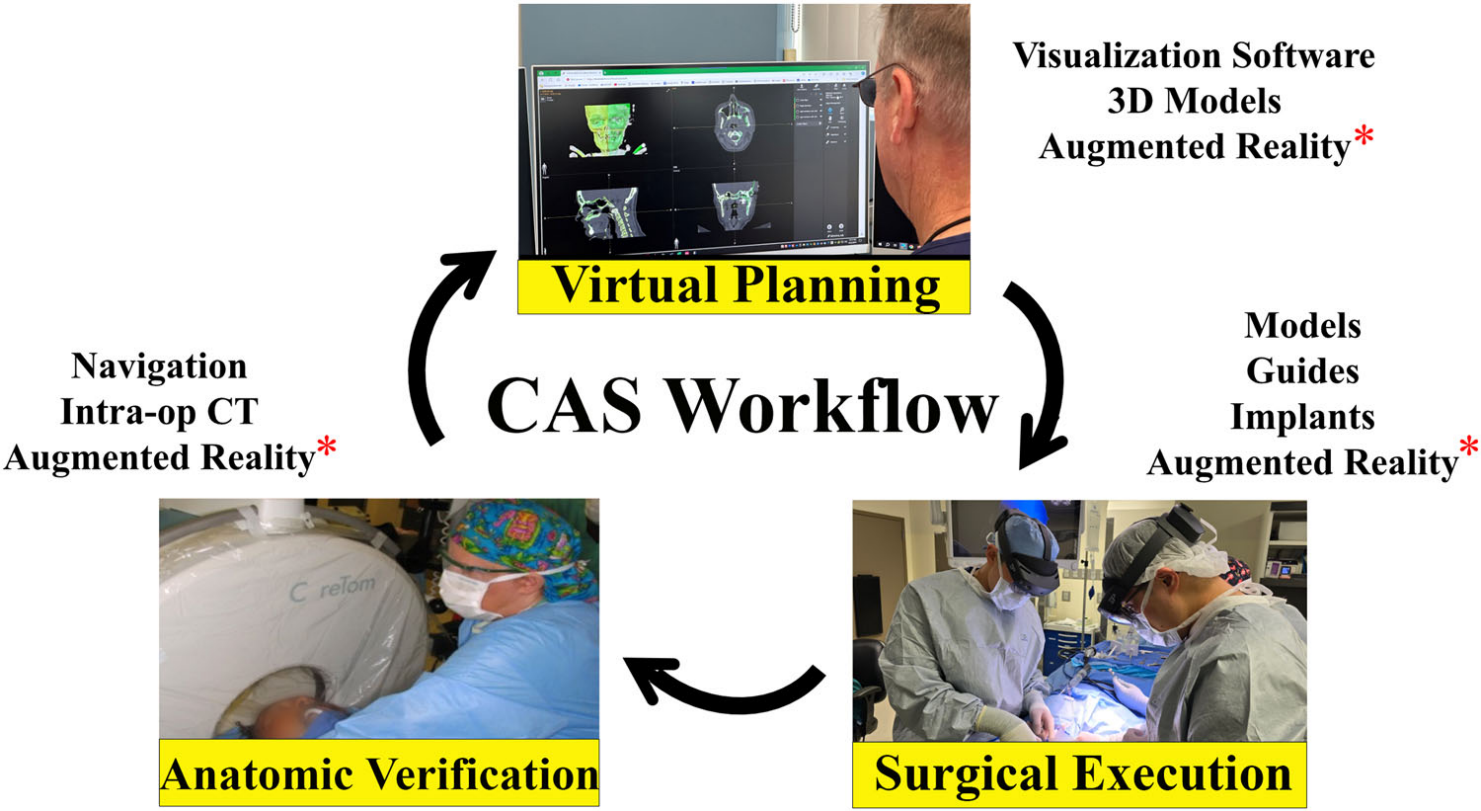
Computer‐aided surgery (CAS) workflow *including virtual planning, surgical execution, and anatomic verification*. *It should be noted that augmented reality is the only application that can be utilized across all the phases of computer‐aided surgery.

AR navigation can be utilized on a number of physical platforms, including HMDs, endoscopes/exoscopes, external projectors, smartphones/tablets, handheld probes, and surgical consoles.^12^ While each platform has their advantages and disadvantages, stand‐alone HMDs and scopes are most feasibly integrated into current intraoperative workflows. Over the past decade plus, extensive research has been pending on the development of AR navigation in CMF surgery; however, the vast majority of the work has been on phantom models,^13^, ^14^, ^15^, ^16^, ^17^, ^18^ cadavers,^12^, ^19^, ^20^ and pre‐clinical models.^12^, ^21^

The clinical application of AR navigation has been substantially rarer, and even when done, there are most commonly significant limitations to these studies, including the lack of any registration system (ie, crude manual manipulation of holographic models),^22^, ^23^ the use of custom software not generally available,^21^, ^24^, ^25^, ^26^, ^27^ extensive use of external camera systems and computers,^12^, ^28^ poor registration accuracy (ie, >1 cm),^29^, ^30^ use of non‐ideal AR platforms (ie, projectors, smartphones, tablets, etc.),^12^ small sample sizes,^10^, ^11^, ^12^ and the surgeon not being the one wearing the HMD intraoperatively.^29^, ^30^

However, in the early 2010s, a research group affiliated with the Shanghai Ninth People's Hospital and Shanghai Jiao Tong University started publishing a substantial body of quality work on the use of AR navigation for mandibular osteotomy/distraction,^24^, ^25^, ^27^, ^31^, ^32^ fibrous dysplasia/craniofacial defects,^33^, ^34^ and orbital hypertelorism.^26^ They laid the groundwork for future AR navigation platforms, but AR technology has significantly improved since the publication of these works with the release of the Microsoft HoloLens and Magic Leap headsets in the late 2010s/early 2020s. Furthermore, the reproducibility of this work is low (or nonexistent) due to the use of complex custom hardware and software, and the need for patient‐specific splints (primarily dental) for holographic registration in all cases. The purpose of this investigation was to evaluate the advantages, disadvantages, and clinical applicability of our newly developed AR platform that offers (1) broad usability in all head and neck procedures, (2) an HMD (Microsoft HoloLens 2) with no external camera systems or computers, (3) a precise registration strategy that is comparable to traditional navigation, and (4) a novel real‐time instrument/bone segment tracking system.

## Methods

The AR navigation platform was collaboratively developed with Xironetic. After obtaining institutional review board (IRB) approval (University of California, Davis IRB# 2236607‐1, David Grant Medical Center IRB# 977005), a retrospective review of all cases utilizing AR navigation between September 20, 2023 and October 20, 2024 was performed. For cases performed at University Hospital Basel and University Hospital Freiburg, this study was conducted in accordance with the principles of the Declaration of Helsinki. Therefore, IRB approval was not required as per local regulations as the study involved only de‐identified, non‐sensitive patient information limited to age, sex, and user feedback. Informed consent was obtained from all patients for their participation in the surgical procedures. Confidentiality and patient privacy were strictly maintained across all sites. The inclusion criteria were all CMF cases employing the AR platform at University of California, Davis, David Grant Medical Center, University Hospital Freiburg, and University Hospital Basel. Exclusion criteria were the cases that did not utilize the AR platform and those in other surgical disciplines (ie, orthopedic surgery, neurosurgery, etc.). Data collected included age, sex, surgical indication, procedure type, procedure location, and documentation of clinical advantages/disadvantages.

### AR Registration

**Figure 2:**
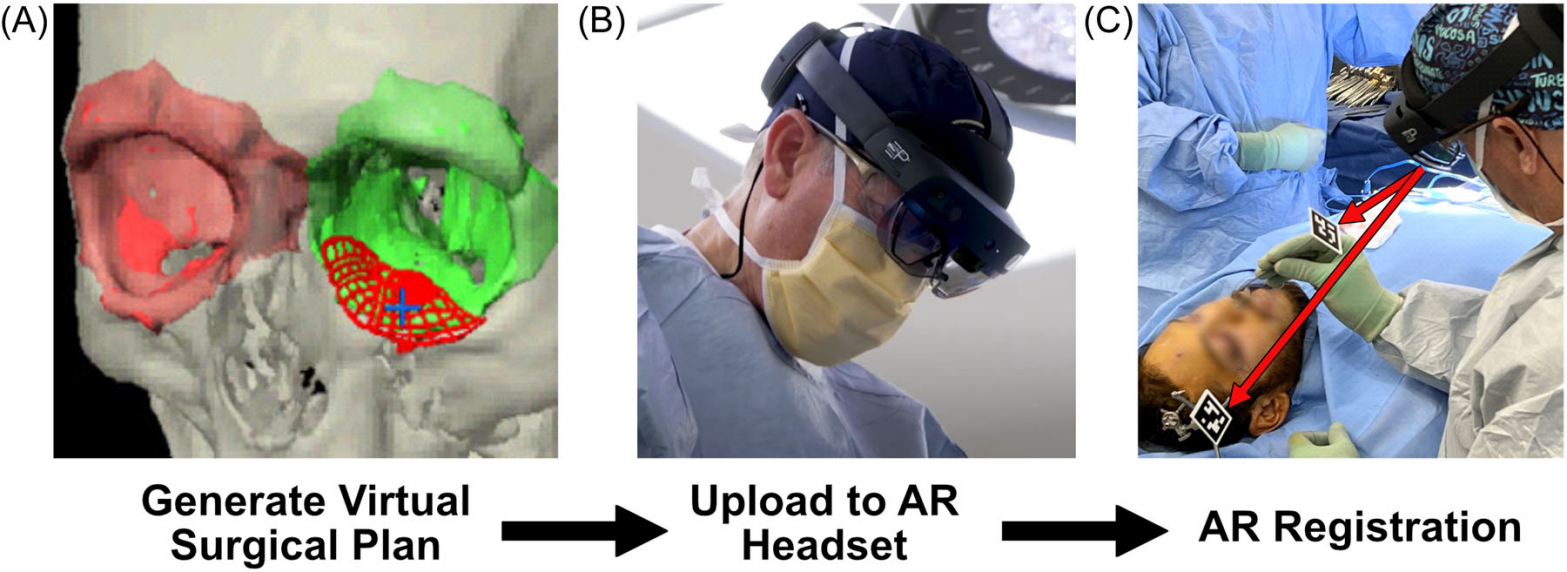
Diagram of the augmented reality navigation workflow. (A) CT/MRI data are used to generate a virtual surgical plan, in this case an orbital reconstruction. (B) The data are then loaded into the AR headset with navigation software (IntraOpVSP by Xironetic). (C) The data set is registered to the patient in the operating room using a stylus with a reflective optical code and a registration anchor fixed to the patient (see arrows).

**Figure 3:**
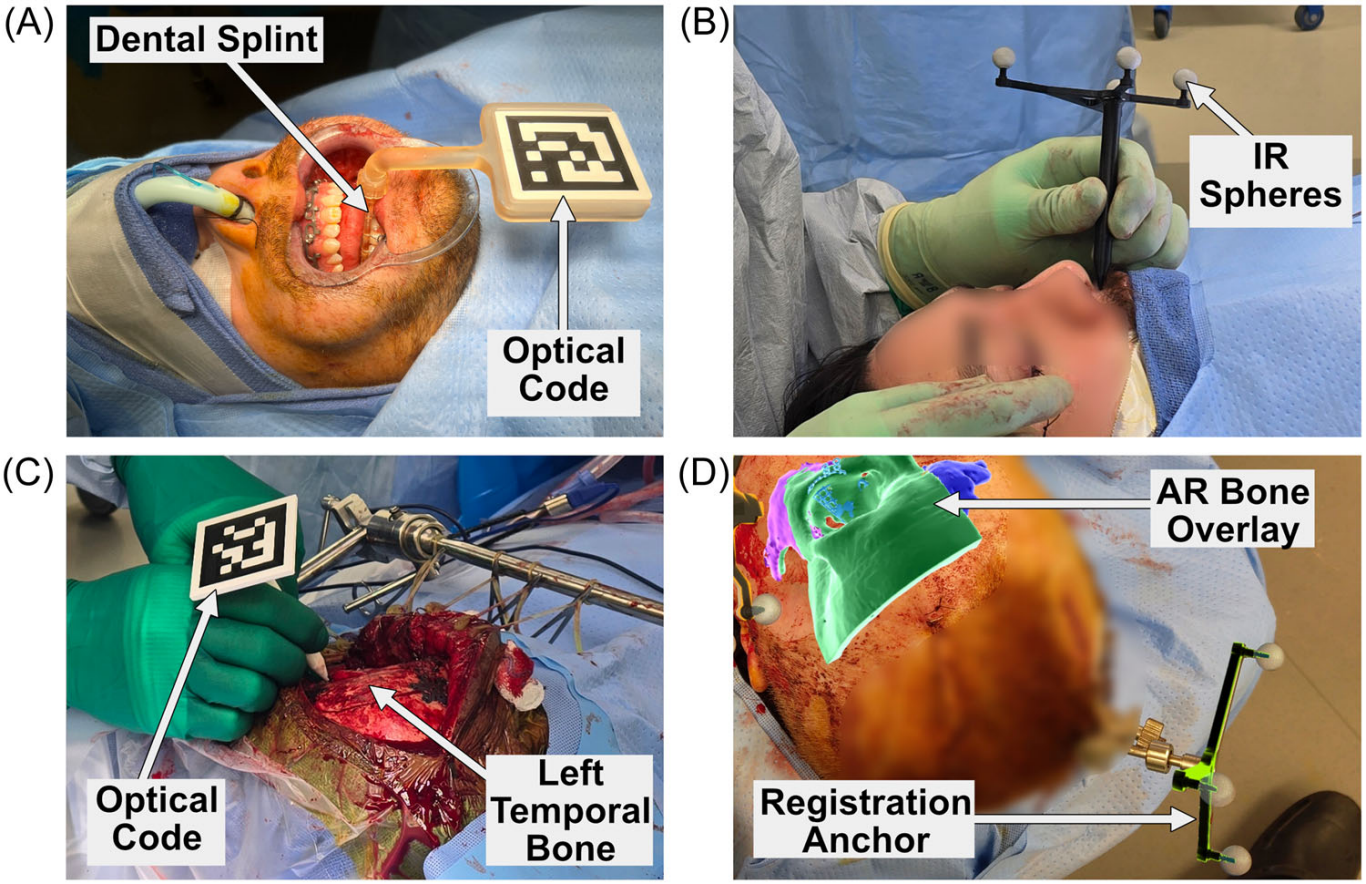
Overview of AR registration methods. The registration process tells the AR navigation headset where the patient is in physical space. (A) AR registration utilizing a patient‐specific dental splint. The spatial relationship between the patient's anatomy and the optical code (black/white ArUco optical code) was preset in the AR headset. (B) Stylus‐based registration with skin‐based anatomic landmarks. In this case, the AR headset used infrared (IR) tracking to follow the stylus. (C) Stylus‐based registration with bony landmarks. In this case, the AR headset used optical tracking of the black and white ArUco optical code to follow the stylus. (D) Image of AR overlay following registration.

Once the patient was prepped, a reference array was affixed to the patient (Figure 2C). The array was typically affixed to the skull, but an oral splint was utilized for most orthognathic cases (Figure 3A). Arrays could also be fixed to the skull with Tegaderm or tape, and not anchored to the bone. The headset was then donned by the surgeon, confirming that both the patient reference array and instrument reference array were visible to the headset. Two different types of arrays were used: (1) infrared (IR) reflective spheres tracked by the IR sensors on the HoloLens 2 (Figure 3B), or (2) an optical code (ArUco marker library—similar to a “QR code”) tracked by the HoloLens 2 optical cameras (Figure 3C). IR tracking provided a larger field of view and better positional accuracy, while ArUco markers had a smaller physical footprint. The skull reference post used for traditional optical navigation (ie, BrainLab) was also used to anchor the AR registration arrays. The surgeon then selected each pre‐determined virtual anatomic landmark on the physical patient. Some cases utilized skin surface landmarks (Figure 3B), and others used bony landmarks (Figure 3C). Landmark placement allowed the AR navigation platform to accurately align the virtual CT/MRI dataset/objects to the patient's physical anatomy. Once registration was complete, the surgeon received immediate feedback on registration accuracy by visualizing the anatomy overlays directly on the patient (Figure 3D). Precise alignment controls were available to fine tune the registration.

### Surgical Navigation/Logistics

Much like traditional navigation, the AR goggles were utilized during specific parts of the procedure where the identification of anatomic/surgical landmarks was critical. While the lens of the HoloLens does rotate up and out of the surgeon's field of view, most surgeons chose to wear the headset only when it was being used. It was donned for patient registration and then re‐applied when AR navigation was utilized. Donning and doffing of the AR headsets was very rapid (less than 1 minute) and performed by an assistant, allowing the surgeon to maintain sterility. There is enough room beneath the lens to wear 2.5 to 3.5 magnification loupes. Some surgeons chose to keep their loupes (or goggles) in place under the AR headset, while others preferred to remove them. Visualization of virtual objects was controlled with voice commands. Each anatomic object was named pre operatively. A list of the objects was continuously projected on drop down menus, high in the field of view, away from the surgical field. The surgeon could refer to the names and use voice commands to make specific objects appear and disappear. For example, the surgeon could say “show zygoma” or “hide zygoma” to make that anatomic structure appear and disappear. Any display modifications were performed using a hands**‐**free protocol. The surgeon could look at a specific drop‐down menu for 2 seconds, which would highlight the menu. Once highlighted, the surgeon looked at the appropriate drop‐down item for 2 seconds to activate it. For example, during the registration process, “registration” was visually selected from a dropdown menu. The headset then visually guided the surgeon through the selection of each anatomic landmark, instructing the surgeon to say “check here” when the probe was on each sequential landmark.

### Trauma

The trauma group included 6 orbital fractures, 3 zygoma fractures, 3 pan‐facial/naso‐orbito‐ethmoid fractures, and 1 hardware removal. The AR platform was utilized for localization of the posterior orbital shelf, implant placement/localization, dynamic localization of the malar eminence, measurement of malar and naso‐orbito‐ethmoid symmetry, and localization of the frontal sinus osteotomies for sinus cranialization.

### Orthognathic

The orthognathic group included 1 Le Fort I osteotomy, 2 bilateral sagittal split osteotomies, 4 bimaxillary advancements, and 1 surgically assisted rapid palatal expansion. The AR platform was utilized for osteotomy placement, visualization of critical structures (greater palatine artery/nerve, mandibular foramen/inferior alveolar nerve, tooth roots), and final bony positioning.

### Tumor

The tumor group included 1 giant cell tumor of the temporal bone, 1 fibrous dysplasia of the zygoma, 1 spheno‐orbital meningioma, 1 skull base hamartoma, and 3 fibular free flaps for squamous cell carcinoma of the mandible. The AR platform was used for osteotomy placement (zygoma and sphenoid bone), and localization of critical structures (optic canal/nerve, superior orbital fissure, stylomastoid foramen/facial nerve, and vasculature structures).

### Craniofacial

The craniofacial group included 2 mandibular distraction procedures, 2 fronto‐orbital advancements, and 1 cranial vault remodeling case. The AR platform was utilized for osteotomy/burr hole placement, avoidance of critical structures (dural sinuses), and final bony positioning.

This case series presents the first‐known examples of (1) AR instrument tracking for midface osteotomies, (2) AR tracking of the zygomaticomaxillary complex during fracture reduction, (3) mandibular tracking in orthognathic surgery, (4) AR fibula cutting guides for mandibular reconstruction, and (5) integration of real‐time IR visualization in an AR headset for vasculature identification.

### Utilization

While quantitative registration and utilization times were not recorded, almost all surgeons felt the user interface was intuitive and had a rapid learning curve. Surgeons uniformly felt the headsets were comfortable and could easily be worn for 20‐minute periods of time.

### AR Navigation Workflow

Thin cut (≤0.625 mm) CT and/or magnetic resonance imaging (MRI) was obtained. Virtual planning was performed utilizing Elements (Brainlab) and 3D Slicer (http://www.slicer.org) (Figure 2A). Typical plans included mirrored anatomy from the contralateral side and/or patient specific implants to be utilized during the procedure. For multi‐modal surgical plans, MRI and CT data were fused in BrainLab Elements. Specific registration landmarks were identified on the patient's scan (ie, virtual patient). Typical landmarks included bilateral tragal notches, bilateral zygomatico‐frontal suture lines, glabella, subnasale, and the inter‐incisal fissure between tooth #8 and #9. The surgical plan was then transferred into the HoloLens 2 (Microsoft) utilizing Xironetic's IntraOpVSP software (Xironetic) (Figure 2B). IntraOpVSP is self‐contained within the HoloLens 2 hardware and requires no external computing equipment. In the operating room, the virtual plan was registered to the patient (Figure 2C) and AR navigation was utilized for the execution and verification of the surgical procedure.

## Results

Thirty‐three cases were performed during the study period (Table 1). The cases fell into 4 categories: trauma, orthognathic, tumor, and craniofacial.

**Table 1:** Table Documenting the 33 Total Patients That Underwent AR‐Navigated Surgical Procedures Across 4 Academic Institutions During the Study Period

	Case	Case					
Case #	Type	Description	Age/sex	Hidden structure visualization	Real‐time tracking	Projected surgical plan	Institution
1	Trauma	Orbit (GSW)	25 M			X	UCD
2	Trauma	Orbit (Assault)	43 M			X	UCD
3	Trauma	ZMC (Fall)	54 M		X	X	UCD
4	Trauma	Orbit (MVC)	79 M	X		X	UCD
5	Trauma	NOE (MVC)	37 M			X	UCD
6	Trauma	Mandibular hardware removal	52 F	X		X	DGMC
7	Trauma	ZMC (MVC)	25 M		X	X	UCD
8	Trauma	Orbit (MVC)	77 F	X		X	UCD
9	Trauma	Orbit (sports injury)	72 M			X	UCD
10	Trauma	Orbit (assault)	27 M	X		X	UCD
11	Trauma	Panfacial (MVC)	29 M	X	X	X	UCD
12	Trauma	ZMC (sports injury)	32 M		X	X	UCD
13	Trauma	Panfacial (GSW)	42 F	X	X	X	UCD
14	Orthognathic	BiMax	21 M	X		X	UF
15	Orthognathic	BiMax	19 M			X	UB
16	Orthognathic	BSSO (assault)	26 M	X	X	X	UCD
17	Orthognathic	Le Fort I (MVC)	37 M	X		X	UCD
18	Orthognathic	SARPE (maxillary hypoplasia)	19 M			X	DGMC
19	Orthognathic	BSSO (mandibular hypoplasia)	34 M	X		X	DGMC
20	Orthognathic	BiMax (Parry‐Romberg syndrome)	34 M		X	X	DGMC
21	Orthognathic	BiMax (severe OSA)	36 M		X	X	DGMC
22	Tumor	Temporal bone (giant cell tumor)	33 F	X		X	UCD
23	Tumor	Zygoma (fibrous dysplasia)	50 M			X	UCD
24	Tumor	Mandible fibula graft (SCCA)	30 M	X		X	UF
25	Tumor	Spheno‐orbital (meningioma)	79 M	X		X	UCD
26	Tumor	Posterior skull base (hamartoma)	2 M	X		X	UCD
27	Tumor	Mandible fibula graft (SCCA)	72 F	X	X	X	UCD
28	Tumor	Mandible fibula graft (SCCA)	63 F	X	X	X	UCD
29	Craniofacial	Posterior vault remodel	5 mo (M)	X		X	UCD
30	Craniofacial	Fronto‐orbital advancement	11 mo (F)			X	UCD
31	Craniofacial	Fronto‐orbital advancement	2 F			X	UCD
32	Craniofacial	Mandibular distraction	1 mo (M)	X		X	UCD
33	Craniofacial	Mandibular distraction	1 mo (M)	X		X	UCD

Abbreviations: BiMax, bimaxillary advancement; BSSO, bilateral sagittal split osteotomy; CVR, cranial vault remodeling; DGMC, David Grant Medical Center; FOA, fronto‐orbital advancement; GSW, Gunshot wound; MVC, motor vehicle collision; NOE, naso‐orbito‐ethmoid; SARPE, surgically assisted rapid palatal expansion; UB, University of Basel; UCD, University of California, Davis; UF, University of Freiburg; ZMC, zygomaticomaxillary complex.

## Discussion

**Figure 4:**
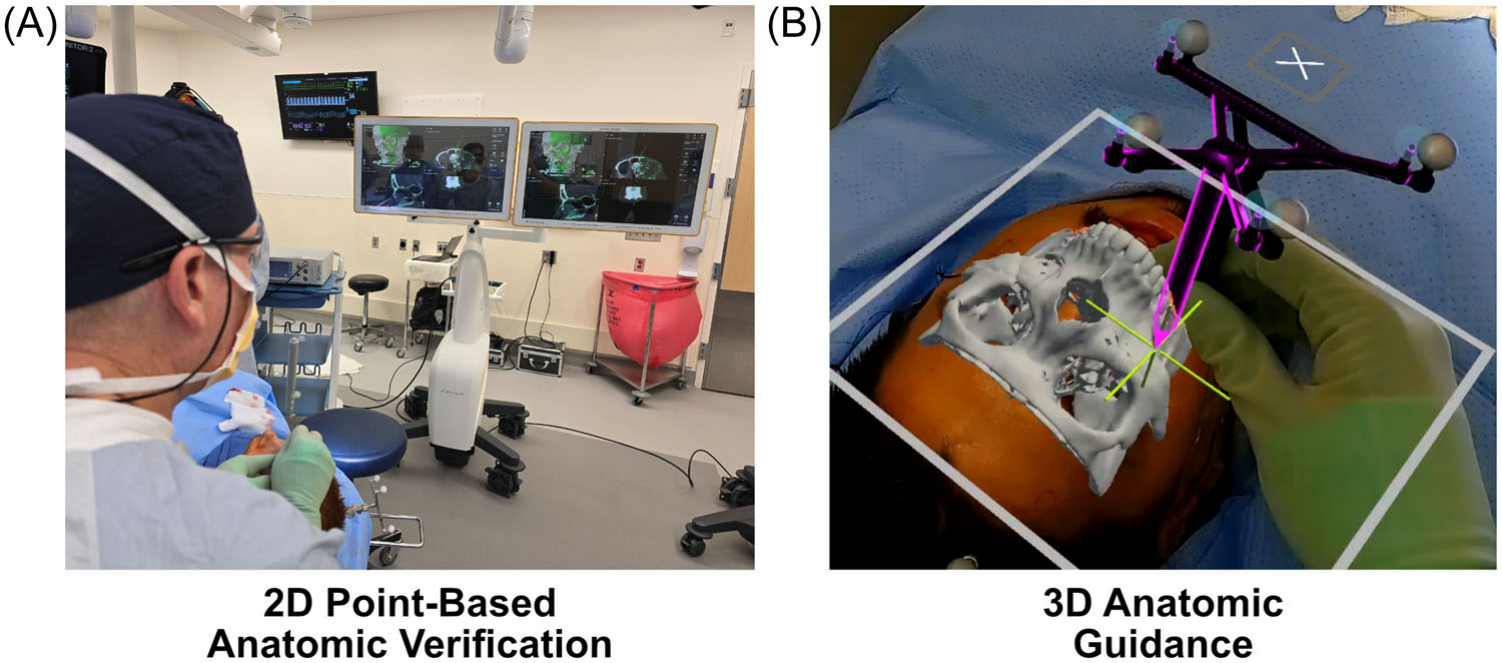
Comparison of (A) traditional 2D, flat panel point‐based surgical navigation versus (B) 3D anatomic guidance provided by AR Navigation.

Traditional intraoperative navigation has been a well‐accepted tool in endoscopic sinus surgery and neurosurgery since the late 1990s. More recently, navigation has been utilized for CMF reconstruction.^35^, ^36^, ^37^, ^38^, ^39^ Traditional navigation utilizes a workstation, a digitizer camera, and a surgical pointer to confirm static anatomic landmarks on a flat panel monitor (Figure 4A). While it is a robust clinical device, it has several limitations including the following: (1) a large footprint in the operating room; (2) a flat panel 2D monitor that provides little dynamic feedback to the surgeon; (3) it requires the surgeon to look away from the surgical field, making it difficult to stabilize the pointer and protect any vital structures in the surgical field; and (4) significant cost. The AR navigation platform excels in many of these areas. The compact AR head‐mounted display allows the surgeon to wear the device, freeing up operating room space. The surgeon can move around the patient, remaining focused on the surgical field, while the AR headset projects and tracks virtual objects (eg, patient specific implant), critical structures (eg, optic canal, vessels, and nerves), and mobile bone segments (eg, zygoma repositioning). The surgeon also does not need to use the pointer to map a large surface area, reducing repetitive and time‐consuming steps compared to traditional navigation. After analyzing all the data, it became apparent that the overarching advantage of the AR platform, compared to traditional navigation, is the real‐time, interactive 3D visualization of the presurgical plan projected onto the surgical field. The platform allows the surgeon to not only visualize specific anatomy but also project virtual objects, readily understand complex anatomic relationships (ie, spatial cognition), and gain situational awareness related to critical hidden anatomic structures at risk in the surgical field (Figure 4B).

These *technical advantages* can be categorized into 3 groups.

### Visualization of the Surgical Plan

**Figure 5:**
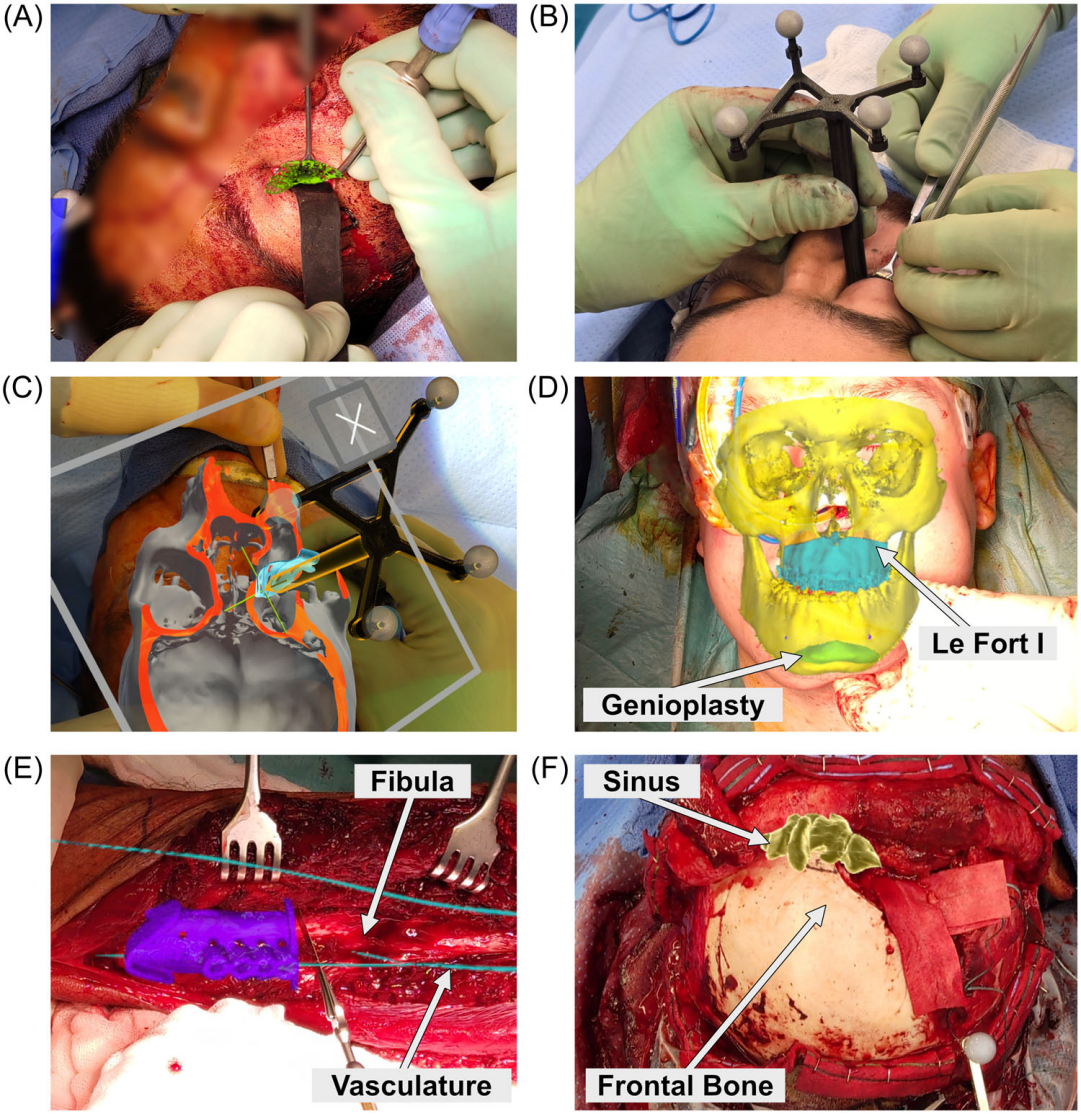
Visualization of the surgical plan. Direct visualization of the surgical plan, projected onto the patient for anatomic guidance, was felt to be a significant advantage for all cases. Some examples include (A) orbital implant visualization, (B) surgical probe placed on the posterior orbital shelf within the orbital cavity, (C) dynamic segmentation of the bony orbit while tracking the position of the probe shown in (B), (D) confirmation of final palatal position after Le Fort I osteotomy and advancement, (E) fibula osteotomy using a virtual cutting guide, and (F) visualization of the frontal sinus for frontal sinusotomy and cranialization.

Direct visualization of the virtual surgical plan, projected onto the patient for anatomic guidance, was a significant advantage for all cases (Table 1). Some clinical examples include (1) visualization of orbital implants (Figure 5A), the posterior orbital shelf (Figure 5B), and final implant position (Figure 5C) during orbital reconstruction; (2) confirmation of the final palatal position after Le Fort I osteotomy and advancement (Figure 5D); (3) fibula osteotomy using a virtual cutting guide (Figure 5E); (4) and visualization of the frontal sinus for frontal sinusotomy and cranialization (Figure 5F).

### Identification of Critical Anatomic Structures

**Figure 6:**
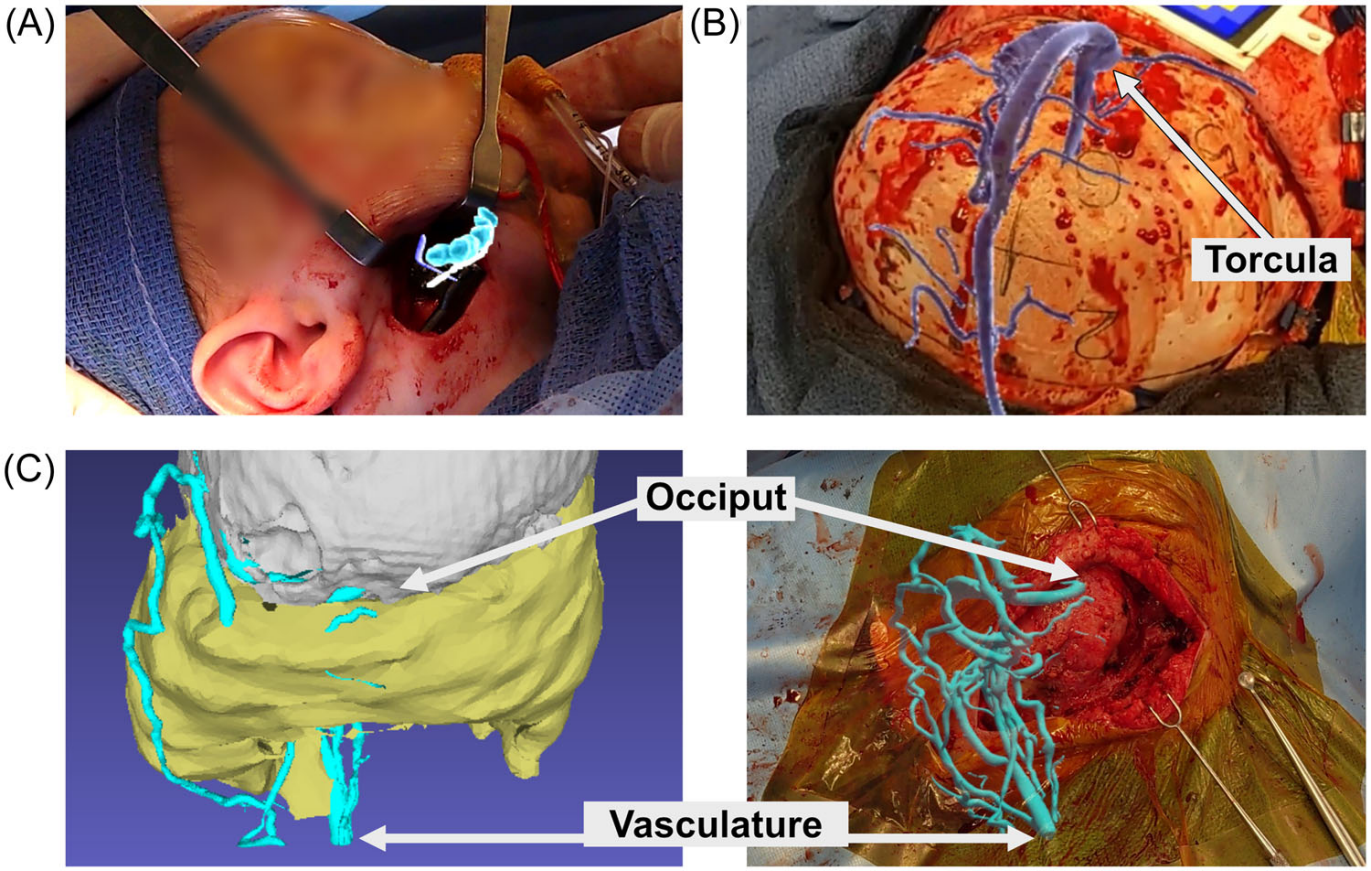
Identification of critical anatomic structures. AR navigation was found to be helpful for identification of hidden anatomy. Some examples include (A) visualization of tooth buds (blue), inferior alveolar nerve (white), and osteotomy plane (purple) during a pediatric mandibular distraction procedure; (B) visualization of an abnormal transverse and sagittal sinus, allowing for safe placement of burr holes during a posterior vault remodeling procedure for a patient with lambdoid synostosis; and (C) visualization of the vasculature during pediatric hamartoma tumor debulking.

**Figure 7:**
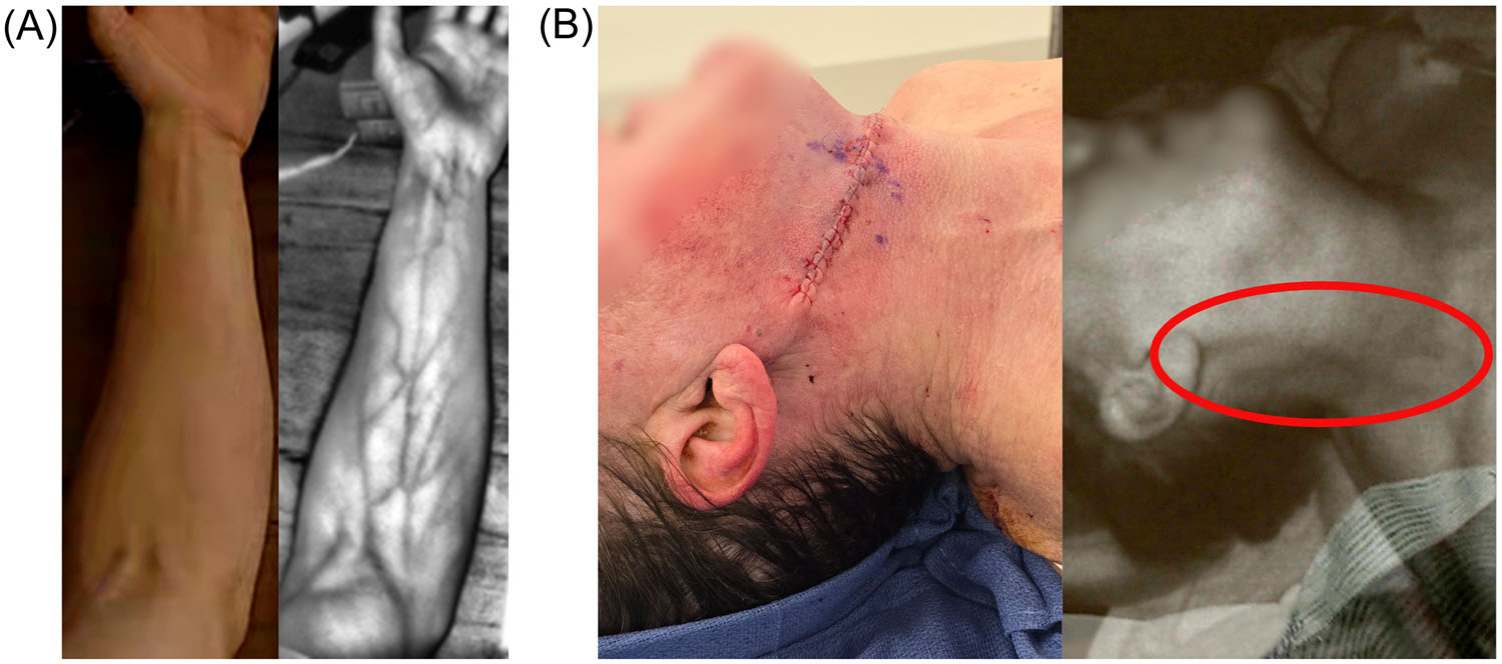
Real‐time visualization of superficial vessels using infrared (IR) sensors on the AR headset. (A) IR visualization of forearm vessels. (B) Photograph of a patient that had recently undergone a free tissue graft for squamous cell carcinoma of the tongue with IR visualization of external jugular vein. This was not visualized with white light alone.

Improved visualization of critical anatomy was felt to be a significant advantage in 19 of the 33 cases (Table 1). Clinical examples include the identification of (1) the tooth buds, inferior alveolar nerve, and osteotomy line during mandibular distraction (Figure 6A); (2) dural sinuses during cranial vault remodeling (Figure 6B); and (3) tumor margins in relation to other vital structures (Figure 6C). Finally, a real‐time IR visualization system was used to dynamically visualize important vasculature during neck dissections (Figure 7).

### Real‐Time Tracking

**Figure 8:**
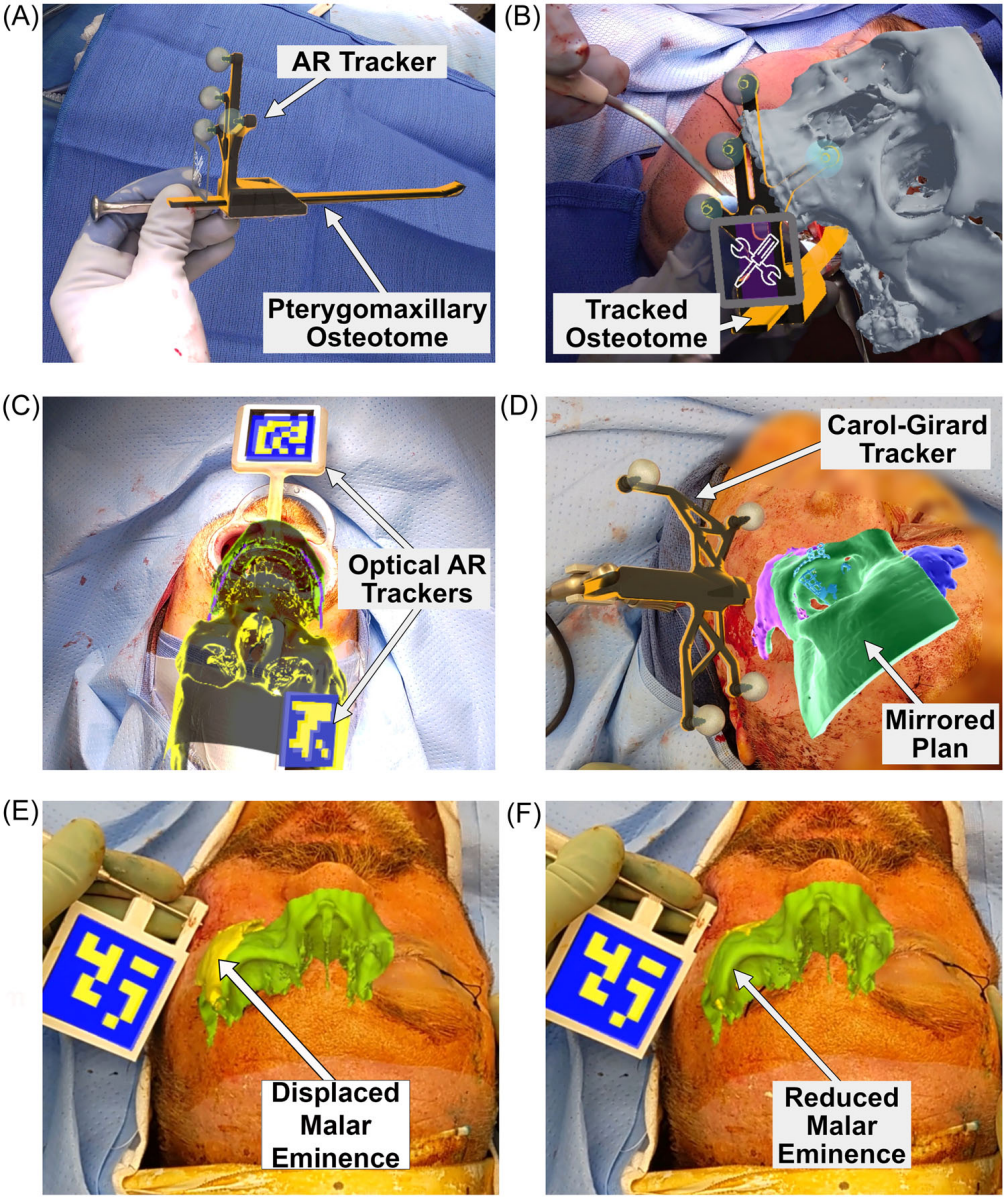
Real‐time tracking using AR Navigation. (A) AR overlay on a tracked osteotome used for pterygomaxillary osteotomy. (B) AR osteotome tracking for pterygomaxillary osteotomy. A virtual clipping plane can be added at the tip of the tracked instrument to give the surgeon a better sense of depth and surrounding anatomic structures. (C) Mandible tracking independent of the cranium for mandibular repositioning in orthognathic surgery. (D) AR navigation of a Carol‐Girard screw for zygomaticomaxillary complex (ZMC) reduction. (E) Closed reduction of a ZMC fracture, utilizing AR navigation to dynamically reduce the malar eminence (yellow) into the correct position on the mirrored anatomic plan (green). (F) Reduced malar eminence.

Real‐time instrument and bone tracking was a significant advantage in 10 of the 33 cases (Table 1). Clinical examples include (1) trackable osteotome (Figure 8A) for tracking of pterygomaxillary osteotomies (Figure 8B), (2) mandibular tracking after bilateral sagittal split osteotomy (Figure 8C), and (3) zygoma tracking for fracture reduction, which includes the placement of a trackable Carol‐Girard screw (Figure 8D) to track displaced bone segment (Figure 8E) into reduced position based on a mirrored anatomy (Figure 8E).

### Study Limitations

Despite the clinical utility of AR in this case series, there are multiple limitations of the technology that need to be addressed. These include *registration accuracy*, *object drift*, *object co‐visualization*, and *overhead lighting.*

**Figure 9:**
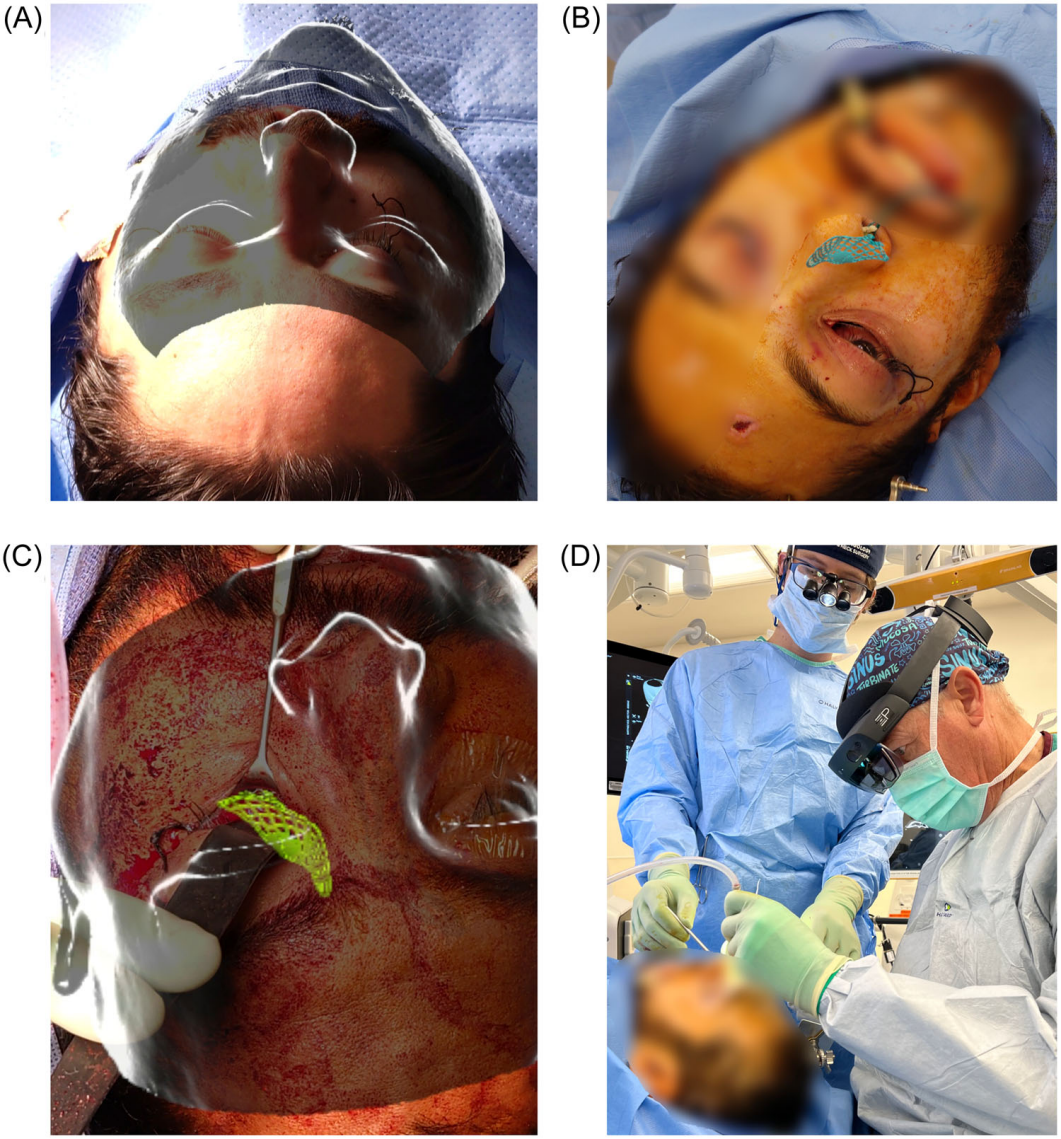
Examples of technology limitations include (A) inaccurate registration due to poor alignment of the virtual dataset onto the patient's anatomy. (B) Object drift results in object movement due to inability of the AR headset to maintain an accurate patient registration during the surgical procedure as shown here with a malpositioning of the virtual orbital implant. (C) This is an example where the opacity of the virtual implant does not allow for co‐visualization of the underlying bony orbital anatomy. (D) There are currently no headlight attachments for the HoloLens; therefore, other light sources must be utilized in deeper surgical fields, as demonstrated here.

*Patient registration* is a complex process requiring an accurate presurgical plan, proper interaction with the user interface, and precise placement of the anatomic landmarks on the virtual model and physical patient. If there are significant inaccuracies in this process, it is possible to see erroneous virtual object placement (Figure 9A). We have already made significant strides in the improvement of registration accuracy (landmark selection, stylus design, stylus tracking methods, etc.) and are actively working to improve registration with new technical advancements, such as point cloud alignment. Furthermore, there is good scientific basis to suggest that AR navigation will ultimately provide superior accuracy to any traditional navigation system on the market currently due to the effective working distance and camera resolution. The IR camera on the HoloLens has comparable resolution to that of current navigation systems, but it is typically positioned at less than one‐third of the distance from the patient. With continued refinement, AR navigation could achieve greater position accuracy in both lab and real‐world conditions.

*Real and apparent object drift* occur when the virtual object appears to move from its anatomic location after an accurate registration. *Apparent object drift* occurs when the surgeon significantly changes their position in relation to the patient. This can result in slight alignment differences which are inherent to the technology but does not result in misalignment of the virtual object in relationship to the registration points. *Real object drift* results in displacement of the virtual object due to the inability of the AR headset to maintain accurate patient registration during the procedure (Figure 9B). Even with apparent and real object drift, the 3D object offers spatial cognition cues that can provide navigational value, which is not the case with 2D navigation. We are actively working to improve stability of object tracking and computational efficiency, as well as improve calibration methods to reduce user perception errors.

*Object co‐visualization* describes the ability to see both the virtual object and the patient's native anatomy. Depending on the ambient lighting conditions and the software algorithm that is used to generate virtual objects, it may be difficult or impossible to see through the virtual object to identify the patient's native anatomy (Figure 9C). We are currently working to improve virtual shaders and tactile feedback to combat these co‐visualization challenges.

*Overhead lighting* is a challenge with AR navigation. The HoloLens 2 was not designed for use with a surgical headlight or overhead operative light. Consequently, surgical procedures that require a headlight for deep cavities are more challenging to perform, and all high intensity lights need to be temporarily removed during 3D visualization (Figure 9D). We are currently working to design a headlight attachment for the HoloLens.

## Conclusion

AR navigation can be utilized in a variety of head and neck surgical procedures including trauma, orthognathic, tumor, and craniofacial surgeries. AR provides distinct advantages over standard navigational tools by providing heads up, real time, interactive 3D visualization of the surgical plan, identification of hidden anatomy, and real‐time segment tracking.

## Author Contributions

**E. Brandon Strong**, study conception, study design, execution, data acquisition, data analysis, data interpretation, manuscript preparation, manuscript review, final approval of the version to be published, accountability for all aspects of the work; **Anuj Patel**, execution, data acquisition, manuscript preparation, manuscript review, final approval of the version to be published, accountability for all aspects of the work; **Alexander P. Marston**, study design, execution, data acquisition, manuscript preparation, manuscript review, final approval of the version to be published, accountability for all aspects of the work; **Cameron Sadegh**, execution, data acquisition, manuscript preparation, manuscript review, final approval of the version to be published, accountability for all aspects of the work; **Jeff Potts**, study conception, study design, execution, data acquisition, manuscript review, final approval of the version to be published, accountability for all aspects of the work; **Darin Johnston**, execution, data acquisition, manuscript review, final approval of the version to be published, accountability for all aspects of the work; **David Ahn**, execution, data acquisition, manuscript preparation, manuscript review, final approval of the version to be published, accountability for all aspects of the work; **Shae Bryant**, study design, execution, data acquisition, manuscript review, final approval of the version to be published, accountability for all aspects of the work; **Michael Li**, study design, execution, data acquisition, data interpretation, manuscript review, final approval of the version to be published, accountability for all aspects of the work; **Osama Raslan**, execution, data interpretation, manuscript review, final approval of the version to be published, accountability for all aspects of the work; **Steven A. Lucero**, execution, manuscript review, final approval of the version to be published, accountability for all aspects of the work; **Marc J. Fischer**, study design, execution, data analysis, manuscript review, final approval of the version to be published, accountability for all aspects of the work; **Marike Zwienenberg**, execution, data acquisition, manuscript review, final approval of the version to be published, accountability for all aspects of the work; **Neha Sharma**, study design, execution, data acquisition, data interpretation, manuscript preparation, manuscript review, final approval of the version to be published, accountability for all aspects of the work; **Florian Thieringer**, execution, data acquisition, manuscript review, final approval of the version to be published, accountability for all aspects of the work; **Christian El Amm**, study conception, study design, manuscript review, final approval of the version to be published, accountability for all aspects of the work; **Kiarash Shahlaie**, execution, data acquisition, manuscript preparation, manuscript review, final approval of the version to be published, accountability for all aspects of the work; **Marc Metzger**, study conception, study design, execution, data acquisition, data analysis, data interpretation, manuscript review, final approval of the version to be published, accountability for all aspects of the work; **E. Bradley Strong**, study conception, study design, execution, data acquisition, data analysis, data interpretation, manuscript preparation, manuscript review, final approval of the version to be published, accountability for all aspects of the work.

## Disclosures

### Competing interests

E. Brandon Strong: Founding Scientist, Xironetic. Anuj Patel: None. Alexander P. Marston: None. Cameron Sadegh: None. Jeff Potts: Co‐Founder, Xironetic. Darin Johnston: None. David Ahn: None. Shae Bryant: None. Michael Li: None. Osama Raslan: None. Steven A. Lucero: None. Marc J. Fischer: None. Marike Zwienenberg: None. Neha Sharma: None. Florian Thieringer: None. Christian El Amm: Founder, Xironetic. Kiarash Shahlaie: None. Marc Metzger: None. E. Bradley Strong: Consultant, BrainLab.

### Funding source

None.
